# Relevance of Pharmacogenomics to the Safe Use of Antimicrobials

**DOI:** 10.3390/antibiotics12030425

**Published:** 2023-02-21

**Authors:** Ann K. Daly

**Affiliations:** Translational and Clinical Research Institute, Newcastle University, Newcastle upon Tyne NE2 4HH, UK; a.k.daly@ncl.ac.uk

**Keywords:** pharmacogenomics, HLA antigens, drug hypersensitivity, chemical- and drug-induced liver injury, antimicrobial agent

## Abstract

There has been widespread implementation of pharmacogenomic testing to inform drug prescribing in medical specialties such as oncology and cardiology. Progress in using pharmacogenomic tests when prescribing antimicrobials has been more limited, though a relatively large number of pharmacogenomic studies on aspects such as idiosyncratic adverse drug reactions have now been performed for this drug class. Currently, there are recommendations in place from either National Regulatory Agencies and/or specialist Pharmacogenomics Advisory Groups concerning genotyping for specific variants in MT-RNR1 and CYP2C19 before prescribing aminoglycosides and voriconazole, respectively. Numerous additional pharmacogenomic associations have been reported concerning antimicrobial-related idiosyncratic adverse drug reactions, particularly involving specific HLA alleles, but, to date, the cost-effectiveness of genotyping prior to prescription has not been confirmed. Polygenic risk score determination has been investigated to a more limited extent but currently suffers from important limitations. Despite limited progress to date, the future widespread adoption of preemptive genotyping and genome sequencing may provide pharmacogenomic data to prescribers that can be used to inform prescribing and increase the safe use of antimicrobials.

## 1. Introduction

Pharmacogenomic testing either preemptively or immediately prior to the prescription of a new drug is an increasingly available option worldwide [1]. However, the implementation of testing remains limited, though it is increasingly being applied in certain medical specialties, especially oncology, cardiology and psychiatry. Antimicrobials are among the most widely prescribed drugs but, at present, the implementation of pharmacogenomic testing in conjunction with their prescription is very limited. The current perspective considers the potential for increasing the use of pharmacogenomic testing when prescribing drugs for the treatment of bacterial and fungal infections. Testing is already used fairly widely when using certain anti-viral drugs [2], but a detailed discussion of this area is outside the scope of the current article.

While many antimicrobials have wide therapeutic windows, which are helpful in terms of safe use, and also show good efficacy when prescribed appropriately, there are particular issues with idiosyncratic adverse drug reactions with some widely used members of this drug class [3]. This aspect and current progress in understanding the underlying basis for susceptibility will be considered in detail, together with examples of recommendations for pharmacogenomic testing in relation to the use of certain antimicrobials.

## 2. Adverse Drug Reactions Involving HLA Gene Associations

Idiosyncratic reactions, particularly involving the skin or the liver (and occasionally both organs), are the most common type of adverse reaction seen for antimicrobials [4]. As reviewed in detail elsewhere [5], there is evidence that many of these reactions are immune-mediated. There are also data pointing to genetic susceptibility to the reactions [6]. The most polymorphic immune-related genes in humans are the human leukocyte antigen (HLA) genes, which contribute to genetic susceptibility to a range of diseases where there is a genetic component. The possibility that a HLA genotype contributes to the risk of idiosyncratic adverse drug reactions has therefore been investigated in detail and a number of specific HLA gene associations involving antimicrobial agents have been reported (Table 1) [6]. Other immune-related genes may also contribute to this risk (see Section 5 below), but, to date, the strongest associations reported have been for the HLA genes.

The reports of strong associations between specific HLA genotypes and susceptibility to adverse drug reactions with antimicrobials summarised in Table 1 have been generally well replicated. This has prompted further studies on both the mechanism for the reaction and the feasibility of genotyping for the risk alleles before prescribing the relevant drug. Good progress has been made in understanding the underlying mechanism, with clear data showing that it likely involves the covalent binding of the drug to cellular proteins followed by an inappropriate T-cell response [20]. Despite the strong and mostly well-replicated associations seen, clinical implementation of genotyping before drug prescription for the associations summarised in Table 1 has not been seen. Even in the case of the strongest association listed (flucloxacillin-induced liver injury and HLA-B*57:01), detailed analysis suggests that genotyping is not cost-effective [21]. It is possible that this may change as the preemptive sequencing of whole genomes with the inclusion of data in electronic medical records becomes more common and data on HLA genotypes are immediately available to prescribers.

## 3. Other Common Adverse Drug Reactions That Appear Independent of HLA Genotype

### 3.1. Anti-TB Drug Treatment and Liver Toxicity

Patients suffering from tuberculosis (TB) are most commonly treated with a combination of the drugs isoniazid, rifampicin, pyrazinamide and ethambutol [3,22]. Mild hepatotoxicity characterised by transient elevation of liver transaminase enzyme levels is common during treatment and affects up to 20% of those treated, as discussed in detail elsewhere [23,24]. However, it is estimated that approximately 1% of those treated with this drug combination suffer significant drug-induced liver injury (DILI) and have more prolonged and severe transaminase elevation combined with other symptoms of hepatitis [25]. These patients usually recover when the drugs are withdrawn but a subgroup may develop liver failure [24]. Most cases of significant liver injury with the anti-TB drugs appear to be due to isoniazid [24,26]. However, rifampicin may contribute to the reaction severity [25], and some cases of DILI due to pyrazinamide alone have also been reported [26]. Patients undergoing treatment with isoniazid alone for latent tuberculosis may also develop DILI [24]. A number of risk factors for the development of DILI due to isoniazid have been identified, including age and some environmental factors, such as alcohol consumption, but genetic risk factors also contribute [24].

**Figure 1:**
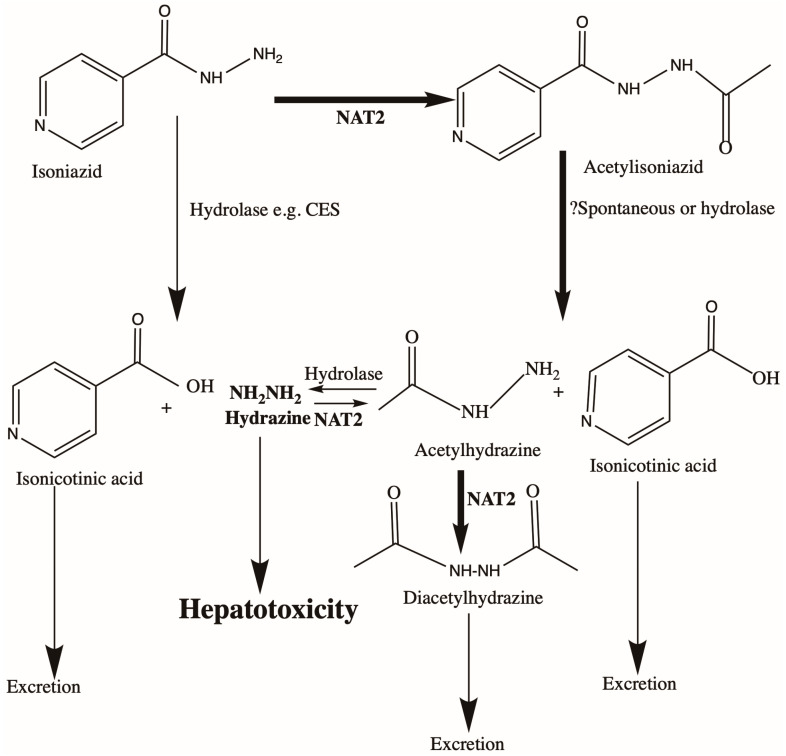
Metabolism of isoniazid by NAT2 and other enzymes. The main metabolites are shown, including hydrazine, which shows hepatotoxicity in animal models.

The most extensively investigated genetic risk factor for the development of isoniazid-related liver injury has been the NAT2 gene, which encodes N-acetyltransferase 2 (NAT2), the major metabolising enzyme for isoniazid (Figure 1). Depending on ethnicity, between 10 and 60% of individuals lack this enzyme activity due to homozygosity for variants causing amino acid changes (for review, see [27]). There is a large body of literature suggesting that NAT2 deficiency is associated with an increased risk of developing DILI, though not all reports agree that this is the case (for review, see [28]). Two recent studies involving genome-wide association approaches suggest that there is a genuine NAT2 association with isoniazid-related DILI [29,30], but one of these [30] found that only those homozygous for variants associated with strongly impaired enzyme activity [31] were at risk. This difference in overall conclusions may be due to ethnic differences in NAT2 allele frequencies and reflect the fact that one study was performed in Thailand and the second in Europe and India. As indicated in Figure 1, the mechanism for toxicity may involve the formation of high levels of hydrazine in those classified as “ultraslow” for NAT2 activity. One study in animals suggested that hydrazine is highly hepatotoxic compared with other metabolites of isoniazid [32]. Though further work is needed in this area, especially on the underlying mechanism for toxicity, results from a clinical trial on assigning an isoniazid dose based on NAT2 genotype are reasonably encouraging [33].

### 3.2. Aminoglycosides and Deafness

Aminoglycoside antibiotics have been known to cause ototoxicity in some patients for many years. There are several different risk factors for this serious toxicity (for review, see [34]), but genetic predisposition is one of these. Genetic susceptibility involves the mitochondrial gene RNR1 (MT-RNR1), which encodes the 12S ribosomal RNA subunit of the mitochondrial ribosome. The basis for this association is now clear, with certain variants showing increased binding of aminoglycoside molecules, which may lead directly to this toxicity within auditory cells [35]. Carriage of one of three variants in MT-RNR1 (1095C > T, 1494C > T and 1555A > G) is considered to be associated with a high risk of aminoglycoside-induced ototoxicity, though there are also a number of other variants where an increased risk is also considered to be possible [36]. Unlike the other gene examples considered in this perspective, the variants relevant to aminoglycoside ototoxicity are very rare. Although the association between RNR1 genotype and susceptibility has been known for some time, recommendations from both National Drug Regulators and a Specialist Pharmacogenomics Advisory Group that testing should be performed in patients prior to aminoglycoside prescription and efforts made to prescribe a suitable alternative antimicrobial to those positive have only recently appeared [37,38]. A recent study has shown the utility of a point-of-care genotyping test prior to gentamycin administration in neonates [36]. This approach can also be applied to adult patients who are also at risk of toxicity if they carry an “at risk” mitochondrial variant [38]. Not all cases of aminoglycoside ototoxicity are due to genetic risk factors, so other risk factors need to be also considered when prescribing these drugs (for review, see [39]).

### 3.3. Fluoroquinolone Toxicity

The fluoroquinolones as a class have been associated with a number of adverse reactions, including skin and liver toxicity, but also a range of other effects, including aortic and tendon ruptures and *Clostridium difficile* infections [40,41]. To date, no associations with HLA genes or indeed any other gene have been reported for any of these reactions. The absence of reports on genetic associations to date may be due to the heterogeneity of the reported reactions and, despite some surveys suggesting adverse reactions to fluoroquinolones are relatively common [40,41], only small numbers of cases are available for detailed analysis. Restrictions on the prescribing of these agents have been recommended since 2018 by the European Medicines Agency (https://www.ema.europa.eu/en/news/fluoroquinolone-quinolone-antibiotics-prac-recommends-new-restrictions-use-following-review, accessed on 27 January 2023), but in view of their broad specificity, understanding better the basis for adverse reactions would be helpful, especially if individuals at increased risk of these reactions can be identified by genetic testing.

## 4. Voriconazole and CYP2C19

Voriconazole is a triazole antifungal agent that is useful in the treatment of invasive fungal infections, particularly aspergillosis [42]. The metabolism of this agent involves a key role for the cytochrome P450 CYP2C19. This gene is subject to common genetic polymorphisms, which can result in either unusually high (ultrarapid metaboliser) or low (poor metaboliser) phenotypes [43], and, in line with this, it is well established that individual plasma concentrations of voriconazole vary considerably [44]. This variability led to a recommendation to perform therapeutic drug monitoring to ensure that adequate levels of the drug are achieved [44]. More recently, it has been proposed by the Clinical Pharmacogenetics Implementation Consortium (CPIC) (a USA-based Pharmacogenomics Advisory Group) that genotyping for CYP2C19 be performed prior to the start of treatment, with the use of alternative antifungals recommended in both ultrarapid metabolisers who have two copies of the CYP2C19*17 allele (approximately 4% of Europeans), and in poor metabolisers who are typically homozygous for CYP2C19*2 (approximately 2% of Europeans) [45]. The ultrarapid metabolisers are unlikely to achieve adequate plasma levels, whereas the poor metabolisers are at risk of a range of adverse drug reactions, including hepatoxicity and visual disturbances. Recent Dutch Pharmacogenomics guidelines (https://www.pharmgkb.org/chemical/PA10233/guidelineAnnotation/PA166104990, accessed on 27 January 2023) suggest that voriconazole dose adjustment based on CYP2C19 genotype may be an alternative approach to the use of other drugs. Voriconazole is the best example of an antimicrobial where a common pharmacogenetic polymorphism in a cytochrome P450 gene affects plasma levels directly and where genotyping for this polymorphism is of considerable value. Access to CYP2C19 genotyping is not always available, even in well-developed healthcare systems, but, as discussed above, therapeutic drug monitoring may be a helpful alternative.

## 5. Polygenic Risk Scores

Many complex traits, including both responses to drugs and susceptibility to adverse drug reactions, are likely to involve contributions from a number of different genetic variants [46,47]. As a result of the considerable progress made in studies involving genome-wide association studies and genome sequencing, a number of polygenic risk scores based on a number of genetic variants have been developed, but very few of these relate to pharmacogenomics [47]. Though a very promising approach, implementation in routine clinical practice to date is limited. At least two studies involving the development of polygenic risk scores to predict susceptibility to drug-induced liver injury due to antimicrobials have now appeared [11,48]. The first of these involved combining data from a genome-wide association study with data from in vitro studies on liver gene expression to select appropriate variants for inclusion in the score [48]. This resulted in the development of a score involving genotypes for almost 28,000 different variants. The genome-wide association study data used to develop this score did not include data from patients with either flucloxacillin- or amoxicillin-clavulanate-induced DILI, and the score was then assessed as a predictor of DILI due to either of these drugs in a new patient cohort. It was found that the score was a statistically significant predictor of both forms of DILI, with higher average risk scores seen in cases of DILI compared with healthy controls. The complexity of the score makes clinical implementation unlikely, but the finding is still interesting because it suggests that there are common susceptibility factors for DILI due to different drugs, unlike the findings with HLA genes discussed in Section 1 above, where different drugs causing DILI tend to show separate HLA associations. It seems likely that the drug non-specific risk factors involve variants, which each contribute a very small increase in risk.

A more recent study on amoxicillin-clavulanate DILI only has led to the development of a less complex polygenic risk score that is more specific for this drug only [11]. This builds on the three HLA associations for DILI due to this drug listed in Table 1. In addition to these HLA associations, two other genetic risk factors for this form of DILI have recently been detected. These are in the immune-related genes PTPN22 and ERAP2 [11,49]. While the effect sizes for the variants in these two genes are lower than those seen with HLA alleles, the two associations have been replicated in an independent cohort [11]. A polygenic risk score for amoxicillin-clavulanate DILI has been developed to include the three HLA risk factors listed in Table 1 (HLA-A*02:01, HLA-B*15:18 and HLA-DRB1*15:01) together with the two additional variants. In a typical population of European ethnicity, 99% of individuals will show a score of 4.5 or lower, with approximately 44% scoring below 0.5. Individuals with a score of 1.58 or higher are at increased risk of developing this form of DILI, with a specificity of 79% and sensitivity of 56%. In the study reporting this finding [11], 56% of the amoxicillin-clavulanate DILI cases had a score of >1.58, compared with 21% of controls. Whether this level of risk makes determining the risk score in advance of prescribing useful is doubtful, but it is possible that the risk score could be used as a diagnostic tool when assessing which drug is responsible for DILI in patients who have been exposed to several different drugs.

## 6. Conclusions

Use of pharmacogenomic tests before prescribing antimicrobials remains limited but, over the last 5 to 10 years, some progress has been made in very specific areas, including the prevention of aminoglycoside ototoxicity and more effective prescription of voriconazole. Both these examples are most relevant to specialist hospital settings, and no pharmacogenomic tests that are suitable for implementation in the primary care prescription of antimicrobials have yet been developed. Although these current examples of implementation are most relevant to specialist hospital settings, the technology required to perform the tests is quite cheap and can be used at the point of care [36,50]. CYP2C19, which is relevant to voriconazole use, is also part of a “direct to the consumer” genotyping panel (https://www.23andme.com/en-gb/test-info/pharmacogenetics/, accessed on 7 February 2023).

When antimicrobials used more widely in primary care and outpatient settings are considered, adverse reactions to these drugs have been well studied at the pharmacogenomic level and interesting associations with HLA and a few other genes have emerged. This has led to a better understanding of the biological basis of these reactions. However, it is currently not cost-effective to implement testing for the risk genotypes described. As both preemptive genotyping and genome sequencing become more widespread [51], this situation may change, with further cost-effectiveness analysis likely to be useful. Refinement and improvement of approaches involving the use of polygenic risk scores is also likely.

## Figures and Tables

**Table 1:** HLA allele risk factors for idiosyncratic adverse reactions related to antimicrobials that affect the skin or liver.

HLA Allele	Type of Idiosyncratic Adverse Reaction	Drug	Odds Ratio (Allelic)	*p*-Value	Reference
A*02:01	Liver injury	Amoxicillin-clavulanate	2.3 (1.8–2.9)	1.8 × 10^−10^	[7]
A*33:01	Liver injury	Terbinafine	40.5 (12.5–131.4)	6.7 × 10^−10^	[8]
B*13:01	Hypersensitivity syndrome (particularly involving skin, with possible liver involvement)	Dapsone	21.7 (10.4–45.1)	2.0 × 10^−16^	[9]
B*14:01-C*08:02	Liver injury	Trimethoprim-sulfamethoxazole	8.7 (3.2–19.5)	2.3 × 10^−4^	[10]
B*15:18	Liver injury	Amoxicillin-clavulanate	3.1 (6.08–1.58)	0.001	[11,12]
B*35:02	Liver injury	Minocycline	29.6 (7.8–89.8)	2.57 × 10^−8^	[12]
B*55:01	Early and delayed allergic reactions (mainly involving skin)	Penicillins	1.4 (1.3–1.5)	2.0 × 10^−31^	[13]
B*57:01	Liver injury	Flucloxacillin	36.6 (26.1–51.3)	2.6 × 10^−97^	[14]
B*57:02 and B*57:03	Liver injury	Anti-HIV and anti-TB comb	30.1 (3.4–263.1)	0.002	[15]
B*57:03	Liver injury	Flucloxacillin	19.8 (3.37–116.1)	0.001	[16]
DRB1*10:01	Immediate hypersensitivity reaction	Penicillins	2.9 (2.0–4.4)	5.4 × 10^−7^	[17]
DRB1*11:04	Liver injury	Nitrofurantoin	4.29 (2.18–7.66)	1.2 × 10^−4^	[18]
DRB1*15:01	Liver injury	Amoxicillin-clavulanate	2.8 (2.1–3.8)	3.5 × 10^−11^	[7]
DRB3*02:02	Delayed hypersensitivity reaction	Penicillins	8.9 (3.4–23.3)	*p* < 0.0001	[19]

## Data Availability

Not applicable.
